# SkiNet: A deep learning framework for skin lesion diagnosis with uncertainty estimation and explainability

**DOI:** 10.1371/journal.pone.0276836

**Published:** 2022-10-31

**Authors:** Rajeev Kumar Singh, Rohan Gorantla, Sai Giridhar Rao Allada, Pratap Narra

**Affiliations:** 1 Department of Computer Science, Shiv Nadar University, Delhi NCR, India; 2 School of Informatics, University of Edinburgh, Edinburgh, United Kingdom; 3 Tandon School of Engineering, New York University, New York, New York, United States of America; 4 Luddy School Of Informatics, Computing, And Engineering, Indiana University Bloomington, Bloomington, Indiana, United States of America; University of Oklahoma, UNITED STATES

## Abstract

Skin cancer is considered to be the most common human malignancy. Around 5 million new cases of skin cancer are recorded in the United States annually. Early identification and evaluation of skin lesions are of great clinical significance, but the disproportionate dermatologist-patient ratio poses a significant problem in most developing nations. Therefore a novel deep architecture, named as SkiNet, is proposed to provide faster screening solution and assistance to newly trained physicians in the process of clinical diagnosis of skin cancer. The main motive behind SkiNet’s design and development is to provide a white box solution, addressing a critical problem of trust and interpretability which is crucial for the wider adoption of Computer-aided diagnosis systems by medical practitioners. The proposed SkiNet is a two-stage pipeline wherein the lesion segmentation is followed by the lesion classification. Monte Carlo dropout and test time augmentation techniques have been employed in the proposed method to estimate epistemic and aleatoric uncertainty. A novel segmentation model named Bayesian MultiResUNet is used to estimate the uncertainty on the predicted segmentation map. Saliency-based methods like XRAI, Grad-CAM and Guided Backprop are explored to provide post-hoc explanations of the deep learning models. The ISIC-2018 dataset is used to perform the experimentation and ablation studies. The results establish the robustness of the proposed model on the traditional benchmarks while addressing the black-box nature of such models to alleviate the skepticism of medical practitioners by incorporating transparency and confidence to the model’s prediction.

## Introduction

Skin cancer is the out-of-control growth of abnormal cells in the outermost skin layer known as epidermis [1]. According to the World Health Organization (WHO), skin cancer accounts for one-third of all cancers, and one out of every five Americans will be diagnosed with it by the age of 70 [2, 3]. There are three main types of skin cancer viz. basal cell carcinoma (BCC), squamous cell carcinoma (SCC), and melanoma. BCC and SCC are the most common forms of skin cancer, with an estimated 4.3 million and 1 million cases reported each year in the US, respectively [4]. BCC and SCC are highly curable, while melanoma is the deadliest form of skin cancer, with 132,000 melanoma skin cancer cases diagnosed worldwide and causing approximately 9000 deaths annually in the US [2, 4–7]. In 2020 alone, 1,198,073 new cases of non-melanoma skin cancer have been reported causing 63,731 deaths [8, 9]. In the year 2021, the number of newly diagnosed melanoma cases in the US is predicted to be 106,110, with 7,180 deaths [10]. In Australia, skin cancer accounts for up to 80% of all newly diagnosed cancers, with two out of every three people diagnosed by the age of 70 [11]. In addition, the rate of non-melanoma and melanoma skin cancers per 100,000 persons in Australia is the highest in the world [11]. In Australia, 16,878 new cases of melanoma were diagnosed in 2021, with 1,315 deaths recorded so far [12]. Internationally, skin cancer also poses a significant threat to public health, with 100,000 new cases of diagnosed melanoma in Europe, and it also accounts for 2-4% of all Asian cancers [2, 13, 14].

Early diagnosis of skin cancer is a cornerstone to combat the rising mortality as the chances of survival drop from 99% to 5% during its progression to the advanced stage [4]. The survival rate of skin cancer is increased by 95% when it is detected early [7, 15, 16]. Conventional clinical approaches such as the ABCD rules [17–19], 3-point checklist [20], and 7-point checklist [21] have been used previously to diagnose skin cancer. However, these strategies are constrained by a number of factors, including the lack of expertise, limited resources, and a lack of time. Further, there is a dearth of dermatologists globally, and in particular, some of the developing nations like Australia and New Zealand suffer from a serious shortage of trained practitioners [5]. With the advent of dermoscopy, a non-invasive imaging technology for providing high-resolution images of lesions, in recent years, clinicians have been adopting it to perform diagnosis. Dermoscopic image analysis by trained medical practitioners achieved clinical accuracy close to 75% [22]. The research community has made significant efforts to develop a computer-aided diagnosis (CAD) system to detect skin diseases from dermoscopy images to provide a second opinion, overcome the limited supply of experts, and provide faster screening solutions. By reducing inter-observer variability and addressing the limited availability of qualified experts, CAD systems strive to improve the performance of human experts in terms of diagnostic accuracy, speed. and reduce the manual inspection [23].

The use of Deep Learning (DL) based CAD tools as a diagnostic aid is a growing trend in dermatology. Further, the advent of Convolutional Neural Networks (CNNs) stimulated the research in various aspects of visual recognition tasks which were highly relevant in the context of medical image analysis [24]. The two building blocks of the CAD system used for this purpose are lesion segmentation and lesion classification. CNNs integrating disease taxonomy were developed to automate both the segmentation and classification task for skin lesion diagnosis. While these systems improved the accuracy significantly, the faith of doctors on these systems did not witness any major upward trend owing to the black-box nature of such CNN based models.

The last decade saw rapid progress of DL research in healthcare across various domains with diverse applications however only a few examples of such techniques are successfully deployed into clinical practice. Medical practitioners must be convinced about the efficacy and accuracy of these systems, however, these models need to suffice at least two primary criteria to gain their trust. The model should have the capability to denote the confidence in each prediction and should be interpretable, i.e., it should clearly represent the features that contributed to the prediction [25]. The model performance is usually presented in terms of metrics related to the discriminative power of the models such as sensitivity, specificity, or ROC curves [26]. However, it is important to understand how confident or certain the model is about a prediction, particularly in clinical practice where diagnostic errors have close to zero tolerance and sometimes difficult cases can require expert review. Estimation of uncertainty can be used not only to determine which samples are difficult to classify, thereby requiring more expert analysis, but also to detect samples that deviate from the data used for model training. The network still can make high assurance predictions when the distribution of training and test data varies. This issue of out of distribution (OOD) sample is an open problem in the domain of DL. Often a crucial problem in the medical setting is deciding whether a model is being used in a environment other than the study.

The machine learning community has traditionally built models that achieve high classification accuracy on a test set, supposedly derived from the same distribution on which the model is being trained. In reality, however, the data the model is being trained on, usually varies from the data on which the model is being deployed [27]. Patients population vary in demographics and in disease presentation between different locations, and these characteristics change with time. Furthermore, datasets are mostly obtained from a few sites with specific procedures for the acquisition of images that may not generalize to other sites [28]. For this reason, it is important to comprehend, how a model makes predictions, beyond optimizing performance on a predefined test set. This provides clinicians with insight as to, when the model will fail. Such intuition enables better model development by targeting data collection to challenge out-of-distribution samples, or by modifying model architectures or by using loss functions to reduce these errors. Moreover, when the model makes a prediction for an inappropriate reason, instead of showing the prediction, the system may refer patients to clinicians. Saliency maps have become a common post-hoc interpretability method for CNNs. These maps are designed to highlight the salient components of medical images which are critical for prediction of the model. This article presents a framework for the segmentation and multiclass classification of skin lesion images by incorporating uncertainty estimation and explainability.The proposed framework called SkiNet will delve into these pertinent issues. The main contributions of the work are as follows:

- Evaluated various methods like U-Net, and MultiResUnet for skin lesion segmentation. Bayesian variant of MultiResUNet is proposed, which provides an uncertainty estimate along with the segmentation map.
- Evaluated the effectiveness of various off-the-shelf CNN models for lesion multiclass classification. We have analysed the performance of Bayesian variants of the top performing classification models.
- Studied the impact of epistemic and aleatoric uncertainty estimation for the top performing classification model.
- Explainability is built into the proposed framework in the form of saliency maps to build the confidence and trust of the medical community in using such models. Comparative analysis of various saliency methods is performed to understand the optimal technique for post-hoc interpretation of skin lesion diagnosis.
- Evaluated the effectiveness of the two-stage SkiNet framework.

The proposed study is beneficial in terms of generalisation and out-of-distribution data since, during training, the interpretability study can clearly aid to identify the important regions that the model is focusing on, rather than the model’s decision being purely coincidental. It aids generalisation in the long term since the model concentrates on the same salient regions even when out-of-distribution data is provided.

This paper is organised as follows. The **Related Works** section provides an overview of various skin lesion segmentation and classification methods. The **Materials and Methods** discusses the methods employed in our SkiNet pipeline along with the dataset used for experimentation. The different metrics used in order to measure the performance of our proposed SkiNet pipeline are discussed in the **Experiments** section. This is followed by the **Results** section, which presents a comprehensive analysis of various experiments of the SkiNet framework and demonstrates the robustness of the proposed framework. The **Discussion** section illustrates the effectiveness of the proposed SkiNet framework. Finally, a brief conclusion and its future scope, is given in the **Conclusion** section.

## Related works

A significant amount of research has been conducted over the past few decades in the field of medical image processing especially for early skin cancer diagnosis. In this section we will first discuss about some traditional techniques that were initially used for skin cancer diagnosis, then we’ll further discuss about the new techniques that were pioneered with the advent of deep learning. We also explore uncertainty estimation and explainable AI, and how they’re changing the landscape.

### Traditional techniques

The earliest CAD system for the diagnosis of skin cancer can be traced back to the late 1980s when researchers used hand-crafted feature extraction techniques based on the ABCD dermoscopy rules, where the skin lesions were characterized based on **A**symmetry, **B**order irregularities, **C**olor distribution, and **D**ermoscopic structures [17, 18, 29]. Border detection, semi-translucence detection, telangiectasia identification, and ulcer/crust detection were among the hand-crafted feature extraction techniques [29]. Lesion segmentation and classification are the two most vital tasks that researchers study to develop CAD systems. The lesion regions are localized and the boundaries of the infected part are drawn in the segmentation task, whereas the localized lesions are classified into the corresponding category in the classification job (i.e., melanoma, benign keratosis, etc). These tasks are challenging due to the variations in the shape, colour, size, and location of lesions, as well as inter and intra-type lesions similarity [10, 30, 31]. Some other factors like low contrast between infected skin lesion pixels and surrounding areas, lesions from different classes having similar signs, and artefacts such as hair, air bubbles, etc., usually act as barriers to segmentation and classification steps [10, 32, 33].

### Deep learning based techniques

Several techniques are introduced in the literature for lesion segmentation and classification. For instance, recently, Bhageri et al. [32] introduced a three-stage segmentation scheme, where in the first stage, off-the-shelf segmentation technique Mask RCNN [34] was used to detect and segment lesions from various modes of an input skin image. In the second stage, a multi-atrous full convolutional neural network was employed to combine the outputs of the Mask RCNN and the input image to present more accurate segmentation results. Finally, in the third stage, a geodesic method was used to modify the boundaries of the lesions. In [2] a DL based saliency segmentation method and CNN feature optimization technique using an improved moth flame optimization algorithm were employed for lesion segmentation and classification. Khan et al. [35] introduced a unique skin lesion detection and classification technique based on probabilistic distribution and feature selection. In the article to segment the lesion region, normal and uniform distributions have been used. The features were then taken from the segmented images and combined using a parallel fusion approach. The entropy-based technique has been integrated with the Bhattacharyya distance and variance formulation for feature selection. Al-Masni et al. [36] proposed a hybrid model for the classification and segmentation of numerous skin lesions. For segmentation of lesion components, a full-resolution convolutional network was used, wherein off-the-shelf deep CNN architectures have been incorporated to classify the segmented skin lesions. Yu et al. [37] designed a hybrid deep learning network with local descriptor encoding wherein deep ResNet features were combined with statistical fisher representations to discriminate between distinct skin lesions using an SVM classifier with a Chi-squared kernel. Recently, Kadry et al. [38] employed VGG-SegNet architecture to extract the melanoma regions from the given dermoscopy images.

Recently [10] introduced a two-stream deep neural network information fusion framework for multiclass skin cancer classification. The proposed method is divided into two parts wherein the first stream, a fusion-based contrast enhancement technique, have been proposed, which feeds enhanced images to the pretrained DenseNet-201 architecture, and features were then improved using a modified moth–flame optimization approach. A finetuned pretrained MobileNetV2 is used in the second stream. Finally, using a new parallel multimax coefficient correlation approach, the most discriminating features from both networks are merged. Khan et al. [39] created a hybrid approach that combined the binary images produced by their proposed 16-layered CNN with a higher-dimensional contrast transform-based saliency segmentation. On the segmented lesion images, a pre-trained DenseNet-201 model was finetuned for classification. After that, using the t-distribution stochastic neighbour embedding (t-SNE) approach, the collected features from the two completely linked layers were down-sampled. Finally, using a multi canonical correlation (MCCA) technique, these features are fused and given to a multiclass Extreme Learning Machine (ELM) classifier. [16] performed segmentation using DarkNet-19 and image fusion-based approach. They extracted features from the segmented masks using DarkNet-53 architecture, and feature fusion was performed using the Parallel Entropy Correlation technique. The softmax classifier was used to predict the lesion class using the entropy kurtosis controlled whale optimizer feature selection technique.

### Uncertainty estimation and explainability

These aforementioned DL techniques over the last decade have improved their performance, however, these methods are black box and lack mechanism for uncertainty estimation and explainability; which are essential in the medical domain. Even though a deep learning model is uncertain about a particular prediction, it would still make a definitive prediction, which might be cataclysmic in the medical diagnosis scenario where there is a very high human, economic, and social cost of error. Displaying a measure of certainty with traditional CAD prediction would allow doctors to adapt their trust according to the model’s confidence. This aspect of certainty and confidence was addressed in recent works like [40], where the stochastic active contour segmentation approach was used to produce a large set of plausible segmentations, and then the weighted sum of these segmentations was calculated to find the uncertain margins. Wang et al. [41] used test time augmentation for measuring uncertainty in the segmentation of MRI scans. Ghahramani et al. [42] proposed the use of dropouts as a Bayesian approximation in order to calculate the model uncertainty. This method was utilized in [43, 44] to measure the uncertainty in classification and segmentation tasks in the medical scenario. Unlike performance metrics such as accuracy, sensitivity, etc., explainability is not entirely quantifiable; however, it is crucial to understand what the model is learning. Recent works [44, 45] have deployed techniques like Guided Backprop and Grad CAM to highlight the essential features that contribute to the DL model’s prediction. This article will attempt to incorporate uncertainity and explainability using a two stage Skinet Framework.

## Materials and methods

### Dataset for lesion segmentation and classification

**Fig 1:**
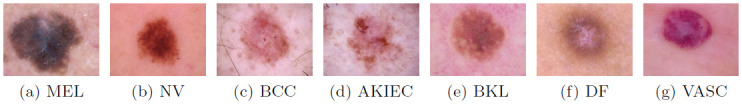
Visual examples depicting the seven categories of pigmented skin lesions.

The ISIC 2018 [46, 47] task 1 dataset has been used in order to train the segmentation model. It consists of about 2594 RGB images and their respective ground truths. The input images were resized to 224×224 with bicubic interpolation and normalized to the [0, 1] range.The classification model was trained and tested on the ISIC 2018 [46, 47] task 3 dataset. The task 3 data contained 10015 dermoscopic images over seven classes viz. Melanoma (MEL), Melanocytic Nevi (NV), Basal Cell Carcinoma (BCC), Actinic Keratoses and Intraepithelial Carcinoma (AKIEC), Benign Keratosis (BKL), Dermatofibroma (DF), and Vascular lesions (VASC) as shown in Fig 1. The dataset suffers from severe class imbalance issues; hence the data was augmented through vertical, horizontal flipping and random rotations in the range of [−65, 65]. The resulting augmentation is an offline augmentation, which contains 13,302 images that have been resized to 450×600. Hence when an image is passed from segmentation output to classification input, it is resized accordingly.

### Methods

In this section we discuss various methods employed in our SkiNet framework. Our proposed SkiNet framework as illustrated in Algorithm 1 is a two-step process (i.e, lesion segmentation and classification) which incorporates the uncertainty estimation and the explainability of the algorithm’s decision. In the first step, we perform segmentation to extract key regions from the input image and then feed this segmented image to our second step which is classification, provided the segment produced is certain or else the original image itself is passed to the second step. If the proposed algorithm is uncertain about its final diagnosis then it would suggest for expert intervention else it would give results with confidence and also show the key pixels which played an essential role in the decision-making process. We have set the uncertainty threshold *φ*_T_ as 0.25 for segmentation and 0.35 for classification which we have arrived at after experimentation to improve model performance.

This subsection discusses the methods for estimating the associated uncertainty and incorporating interpretability in our model. Further, it describes segmentation and classification techniques suitable to the task at hand.

**Algorithm 1** SkiNet framework/pipeline

1: **procedure** Given image *I*, Segmentation model *M*_*s*_, Classification model *M*_*c*_

2:  Pass *I* through *M*_*s*_

3:  Get segmented image *S* and estimate uncertainty metric *φ*

4:  **if** *φ* < *φ*_T_ **then**     ▹ *φ*_T_ = 0.25 for segmentation

5:   Pass *S* through *M*_*c*_     ▹ *S* certain

6:  **else**

7:   Pass *I* through *M*_*c*_     ▹ *S* uncertain

8:  Get predicted class *P* and estimate uncertainty metric *φ*

9:  **if** *φ* < *φ*_T_ **then**     ▹ *φ*_T_ = 0.35 for classification

10:   *I* is diagnosed as predicted class *P*     ▹ *P* certain

11:  **else**

12:   Go for physician’s second opinion     ▹ *P* uncertain

13:  Get Explainability map *X*

#### Methods for uncertainty estimation

CNNs have some shortcomings despite their progress in a wide range of applications. One of the concerning drawbacks among them is its inability to provide a notion of uncertainty in its prediction, which is crucial in the medical domain [48]. For example, in a case where the CNN model was trained on a range of car data in order to predict the category to which the given car belongs, the hypothetical model should return a prediction with a high level of confidence. But what if the model is validated with a bike image and asked to choose a car category? This is a situation where the test data is far from the distribution as the model is trained on distinguishing among various car classes and have never seen the image of a bike. In such cases, the model is expected to return a prediction and some additional details communicating the high degree of uncertainty with these kinds of data. Uncertainty estimate can be used to assess samples which are difficult to identify, thereby requiring a further expert review, and to detect samples that deviate from the data used to train the model.

There are mainly two types of uncertainty viz., Aleatoric and Epistemic uncertainty [49]. Aleatoric uncertainty captures noise inherent in the data and cannot be abated by collecting more data [50]. Epistemic uncertainty, also known as model uncertainty, accounts for variability in the parameters of the model and analyzes what the model is not aware owing to the lack of training data [42]. Epistemic uncertainty is helpful to understand examples that vary from training data especially in situations where we have small and imbalanced datasets, which is common in CAD systems [50].

Uncertainties are formulated as probability distributions over the model parameters (for epistemic uncertainty) or model inputs (for aleatoric uncertainty) [42]. Bayesian statistics have largely inspired most of the work done till now on uncertainty estimation techniques. Bayesian Neural Network (BNN) [51] is the probabilistic variant of the traditional neural networks and provides a mathematical framework for uncertainty estimation. Most of the earlier works on epistemic uncertainty estimation are based on Bayesian inference. However, in practice, Bayesian inference is computationally expensive; therefore, extensive research has been done in developing various techniques to approximate Bayesian deep networks although they are not scalable for larger convolutional networks [52–55]. Research has also been carried out to develop alternative strategies, which are suitable for approximating the uncertainty [42, 56]. The work proposed by [42] demonstrated how dropout [57] applied on a neural network with an arbitrary number of layers is mathematically equivalent to estimating variational inference in Gaussian process model [58]. This was later extended to CNNs in [59] explaining that dropout can be used to enforce a Bernoulli distribution over the weights of the CNN without any additional model parameters. This method is known as Monte Carlo (MC) Dropout and is successfully employed in some of the applications in the medical imaging domain [26, 60].

The dropout layers are generally added in many deep neural networks to reduce overfitting by randomly dropping weights with a fixed probability. Inspired by the capability of the MC-Dropout technique in estimating uncertainty, we employ the same to build our proposed model. Given a test sample s^*^, we sample the network *B* times over its parameters and thereby giving an estimate of the predictive posterior distribution. This sampling is known as Monte Carlo sampling and the mean *μ*_*e*_ over these iterations is considered as the final result on a given test sample. *μ*_*e*_ is computed as shown in the equation below [42]

$$\begin{array}{c} \mu_{e}\approx \frac{1}{B}\sum_{m=1}^{B}p\left(y^{*}|s^{*},{\hat{W}}_{B}\right) \end{array}$$ (1)

where ${\hat{W}}_{B}$ denotes the weights of the network with dropouts in *B*^*th*^ MC iteration and *B* is the total number of sampled sets of weights. Among several classes y*, the one with *μ*_*max*_ is selected as the outcome for each test sample s*.

Aleatoric uncertainty captures noise inherent in the data and cannot be abated by collecting more data [50]. Aleatoric uncertainty can be estimated either by learning a mapping directly from the input data [50] or by test-time data augmentation [41, 61, 62]. However, the former technique suffers from the drawback, as is it involves adapting the network architecture and loss function, which restricts the application to trained models. Therefore, we employ test-time data augmentation technique in our pipeline. In this approach, a test sample s* is augmented to form *V* different versions of the image and is forwarded to the network. The mean *μ*_*a*_ over these iterations is considered the final result of a given test sample. *μ*_*a*_ is computed as shown in the equation below [50]

$$\begin{array}{c} \mu_{a}\approx \frac{1}{V}\sum_{v=1}^{V}p\left(y^{*}|s_{v}^{*},\hat{W}\right) \end{array}$$ (2)

where $s_{v}^{*}$ denotes the *v*^*th*^ augmented image, $\hat{W}$ denotes the weights of the network and *V* is the total number of image augmentations. Among several classes y*, the one with *μ*_*max*_ is selected as the outcome for each test sample s*.

These two approaches are then combined to calculate the overall uncertainty where a test sample s* is augmented to form *M* different versions of the image and is forwarded to the network with the dropout activated during the test time. The mean *μ* over these iterations is considered as the final result on a given test sample. *μ* is computed as shown in the equation below [41, 61]

$$\begin{array}{c} \mu \approx \frac{1}{M}\sum_{m=1}^{M}p\left(y^{*}|s_{m}^{*},{\hat{W}}_{m}\right) \end{array}$$ (3)

where $s_{m}^{*}$ denotes the augmented image passed and ${\hat{W}}_{m}$ denotes the weights of the network with dropouts during the *m*^*th*^ iteration and *M* is the total number of iterations. Among several classes y*, the one with *μ*_*max*_ is selected as the outcome for each test sample s*.

In order to estimate the model uncertainty *φ*, we calculate the entropy of the averaged probability vector across the *N* classes using the equation as given below [41, 61, 62]

$$\begin{array}{c} \varphi =-\sum_{n=1}^{N}p_{n}\text{log}p_{n} \end{array}$$ (4)

here *p*_*n*_ is the probability of *n*th class.

#### Methods for explainability

CNNs lack interpretability, which is an essential requirement in the medical domain due to the possibility of life-threatening consequences. A medical practitioner needs to understand the key features in the image used by the given model to make predictions to verify if it is consistent with medical knowledge and build trust in the model’s capability. While interpretability is desirable in all domains, since medical practitioners have to deal with medico-legal, ethical, and strict regulations it becomes all the more essential in the medical domain. Recently, there has been considerable research on saliency methods that relate CNNs prediction to the inputs that have maximum influence on the prediction. These techniques may be useful in a variety of ways, including tracking a model’s assessment, ensuring that the model does not learn false correlations, and evaluating the model for issues related to fairness [63, 64].

Saliency based methods can broadly be classified into two categories. One collection of methods modifies the input and computes the effect of this change on the output by making a forward pass through the network using these altered inputs [65, 66]. The other set of approaches calculate attributions by returning the prediction score back to the input features through each layer of the network. In general, second category methods are faster than the initial set of methods, as they usually require a single or constant number of neural network queries [67]. Guided Backprop [68], Grad CAM [64], Guided GradCAM [64] and XRAI [67] are some of the promising approaches in this category. Therefore we explore these techniques in our approach to bring model interpretability in the context of skin lesion detection.

**Guided Backpropagation** is a technique for visualizing CNN by slightly modifying the backpropagation algorithm wherein the negative gradients are set to zero in each layer, allowing only positive gradients to flow backwards through the network. Guided Backpropagation is a combination of Backpropagation and deconvolution. During forward pass, due to the presence of ReLU activations, all the negative input values passed through neurons are set to zero. Therefore, during the backward pass of Backpropagation, the gradients don’t flow back through these neurons. In deconvolution, during the backwards pass all the negative gradients are suppressed to zero. In guided Backpropagation, both the negative gradients and the gradients with negative input are suppressed to zero. The rationale behind this modification is that all the positive gradients of higher magnitude imply key pixels, while negative gradients denote the pixels the model wants to suppress.

**Grad-CAM** provides a visual explanation by leveraging the gradient information coming into the final convolutional layer. The last layer is chosen as it provides the best tradeoff between detailed spatial information and high-level semantics [64]. It considers the convolutional layer since the convolutional features generally possess spatial information. The key pixels responsible for categorising a particular class are determined by forward propagation through the network by obtaining gradients for each class. The gradients during backpropagation are average-pooled to obtain the weights that are important for the target class prediction. The weights obtained are combined with activations maps using ReLU operation to compute the Grad-CAM heatmap. To generate a Grad-CAM heatmap $V_{Grad-CAM}^{e}\in R^{w\times h}$, of width *w* and height *h* for a class of interest *e*, the gradient of the score of class *e*, *z*^*e*^ is calculated with respect to the feature maps *F*^*a*^ i.e$\frac{\partial z^{e}}{\partial F^{a}}$. Then these gradients are global average pooled to get the neuron importance weights $\beta_{a}^{e}$ using the equation given below [64]:

$$\begin{array}{c} \beta_{a}^{e}=\frac{1}{G}\sum_{k}\sum_{l}\frac{\partial z^{e}}{\partial F_{kl}^{a}} \end{array}$$ (5)

$\frac{1}{G}\sum_{k}\sum_{l}$ correspond to the global average pooling. The $\beta_{a}^{e}$ represents a partial linearization [64] of the network downstream from *F* for a class of interest *e*.Finally to obtain the heatmap, a weighted combination of forward activation maps is computed followed by a ReLU as given below [64].

$$\begin{array}{c} V_{Grad-CAM}^{e}=ReLU(\sum_{a}\beta_{a}^{e}F^{a}) \end{array}$$ (6)

**Guided Grad-CAM** overcomes the drawback of Grad-CAM, which is the inability to show fine-grained importance like Guided Backpropagation, a pixel-space gradient visualization method [64]. Guided Grad-CAM is the blend of Guided Backpropagation and Grad-CAM algorithms via pointwise multiplication to incorporate the advantages of both methods. First, the heatmap $V_{Grad-CAM}^{e}$ of an input image is obtained via Grad-CAM. Then, this heatmap is upsampled to the input image resolution. Finally, it is pointwise multiplied with Guided Backpropagation to get Guided Grad-CAM visualization. The resultant visualization has high resolution and is class discriminative.

**XRAI** is the most recently proposed saliency method based on Integrated Gradients [69] that decides the key inputs by changing the network input from baseline to the original input and consolidating these gradients. It begins by segmenting the image using Felzenswalb’s graph-based segmentation [70] technique, followed by repeated testing of the significance of each segment using attributions. Integrated gradients are used as attribution with black and white baselines to resolve their setback as they are insensitive to pixels similar to or equal to the baseline image. Thus, every pixel gets an equal chance to contribute to the attributions regardless of the distance from the baseline. Finally, it merges regions with a higher positive value of the sum of all the attributions of that region until it has the complete image as the mask or runs out of regions to add [67].

#### Methods for segmentation

In medical image analysis, some pixels in the image contain vital information that might play a crucial role in decision-making, thereby providing a rationale for the treatment. Segmentation would help in augmenting the classification model performance in most cases and, furthermore, would reduce the computation time [71]. In the latter part of the last decade, CNN based segmentation algorithms performed well in biomedical image segmentation tasks. More importantly, U-Net [72] has emerged as one of the most promising architecture in this domain and has been applied to various image segmentation tasks [73–75].

U-Net defined the state of the art in the medical image segmentation tasks [76], however it is not robust enough to analyze objects in the image present at different scales. One of the novel ideas of U-Net architecture has been the implementation of shortcut links between the corresponding layers before the max-pooling and after the deconvolution operations, to relay the spatial information that gets lost from encoder to decoder during the pooling process. The dispelled spatial features though retained, still suffers from shortcomings in the skip connections i.e., there is a plausible semantic gap between the two sets of features being merged. The features from the encoder are supposed to be lower-level features, and on the contrary, the decoder features are of much higher level because they come from deeper layers after fairly complex computation [77].

**Fig 2:**
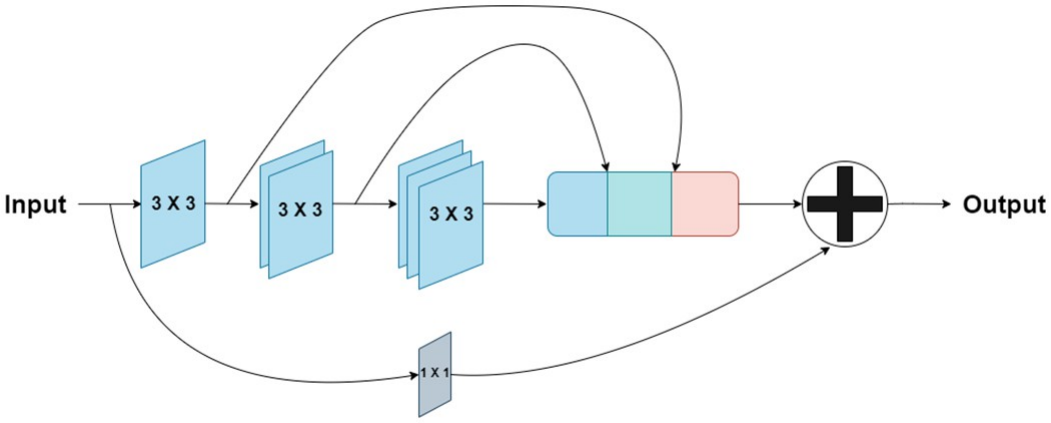
MultiRes block: The rounded rectangle represents a concatenation operation where the black block represents a 3 × 3 convolution, the green block represents a 5 × 5 convolution and the red one represents a 7 × 7 convolution. Finally a skip connection is added along with 1×1 filter.

**Fig 3:**
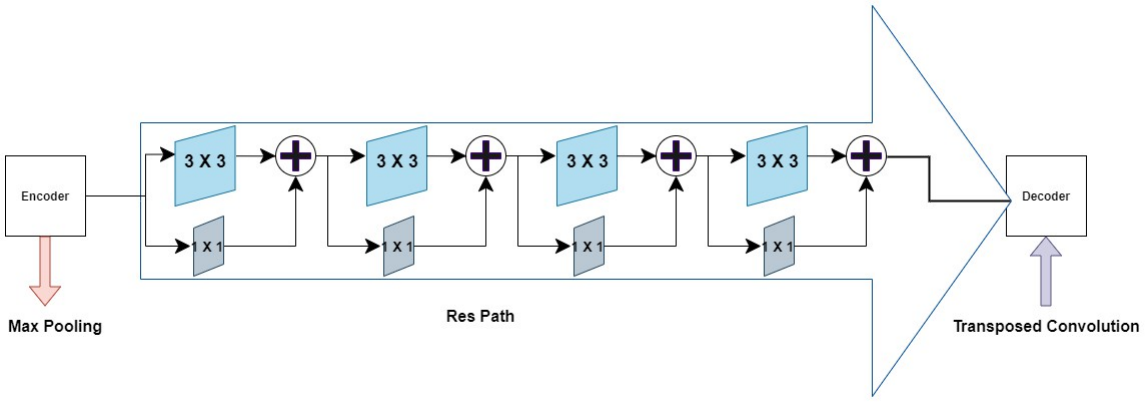
Res path: The encoder features are passed through a series of convolutions instead of linearly connecting them to the decoder features.

In order to tackle the shortcomings discussed above, [77] proposed few structural changes in the form of *‘MultiRes block’* and *‘Res path’* to the U-Net architecture drawing inspiration from [76, 78, 79]. Inspired by the sucessful working of MutiRes block and Res path structures, we employ it in our segmentation architecture known as *Bayesian MultiResUNet*. Similar to the Inception blocks [80], where convolutional layers of different kernel sizes are adopted to inspect the points of interest in images from different scales, MultiRes blocks employs 3 × 3, 5 × 5 and 7 × 7 filters in parallel with the larger and computationally expensive 5 × 5 and 7 × 7 blocks factorized as a succession of 3 × 3 without affecting the objective function [78]. Additionally, MultiRes blocks contain 1 × 1 convolutional layers, for better comprehension of spatial information as shown in Fig 2. Rather than just concatenating the feature maps from the encoder stages to the decoder stages as in the shortcut connection of U-Net, Res paths transfers them through a chain of convolution layers with residual connections and then concatenates them with the decoder features to mitigate the gap between encoder and decoder features. Res path is represented in Fig 3 below.

**Fig 4:**
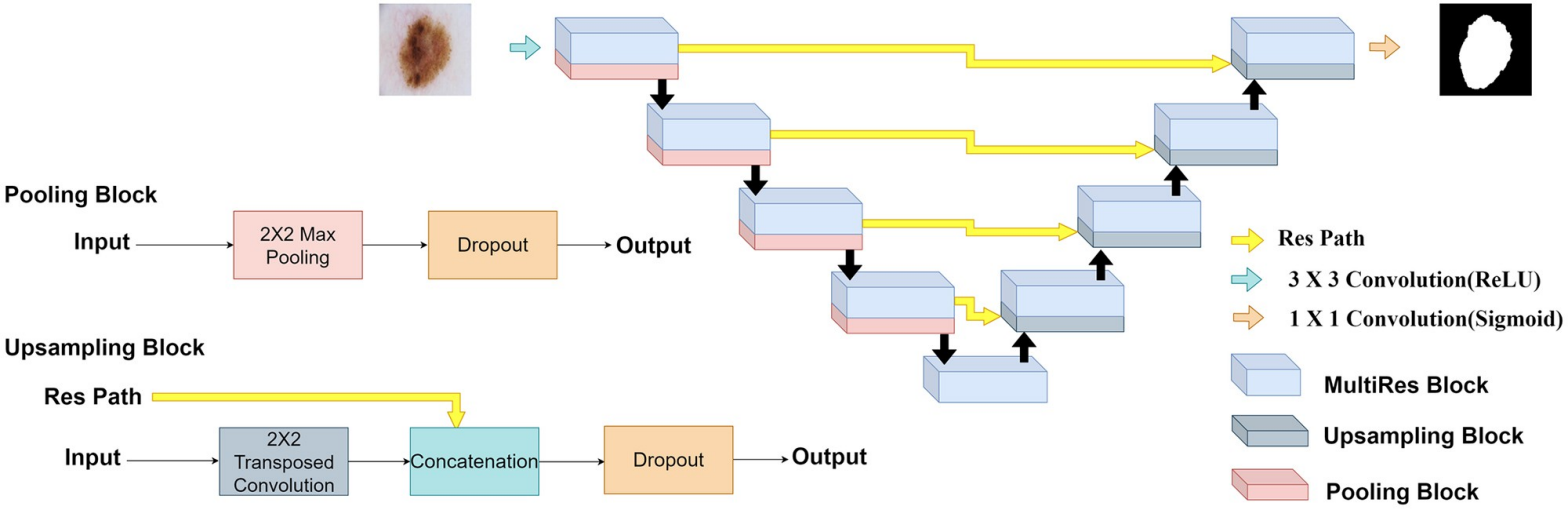
Bayesian MultiResUNet comprises an encoder and a decoder pathway, with skip connections and dropout layers between the corresponding layers in pooling and upsampling blocks.

As depicted in Fig 4, Bayesian MultiResUNet has symmetric architecture where the encoder is responsible for extracting spatial features from the input image while the decoder produces the segmentation map using the encoded features. In the encoder, the weights obtained from the MultiRes block are passed to a pooling block where a dropout layer is appended after the pooling operation and these acquired weights are used as an input to the next MultiRes block. The fifth MultiRes block acts as a bridge between encoder and decoder with three 3 × 3 convolution operations followed by one 1 × 1 convolutional operation. On the other hand, the decoder begins at the upsampling block, which incorporates 2 × 2 transposed convolution operation [81] to perform upsampling thereby reducing the feature channels by half. These weights are then passed on to the MultiRes block, similar to the encoder. This succession of upsampling and MultiRes operations is repeated four times, reducing the number of filters by two at each stage. Finally, a 1 × 1 convolution operation is performed to generate the segmentation map. As we step towards the inner shortcut routes, the intensity of the semantic gap between the encoder and the decoder function maps would possibly decrease; thus we reduce the number of convolutional blocks, i.e., we employ 4, 3, 2, 1 convolutional blocks respectively along the four Res paths. We use 32, 64, 128, 256 filters in the blocks of the four Res paths respectively to compensate for the number of feature maps in encoder-decoder similar to [77]. ReLU [82] activation function and batch-normalization [83] are employed by all convolutional layers in this architecture, except for the final one which uses a Sigmoid activation function.

#### Methods for classification

In our experimentation, we initially trained various off-the-self Classification architectures like Inception [80], Xception [84], VGG-19 [85], DenseNet-169 [86] and ResNet-50 [87]. Furthermore, we have selected the top two models and obtained the Bayesian version of these networks by adding dropouts. The dropout can even degrade the performance of the model; therefore we empirically evaluate the performance of several Bayesian models with various configurations, which include the positioning of dropout layers as well as the dropout rate, to identify those with the best performance of prediction for the skin lesion classification task. Moreover, all the Bayesian networks employed in our analysis are approximate Bayesian models, as the exact Bayesian inference for neural networks is computationally intractable.

## Experiments

### Experimental setup

For the purpose of experimentation we have made use of a cloud based Nvidia RTX 2080ti GPU. The segmentation models i.e the U-Net and Bayesian MultiResUNet were trained with a learning rate of 10^−3^ which we have arrived at after suitable experimentation and a batch size of 16 for better generalization. Different dropouts ranging between [0.4,0.7] were applied to get the best model which would not overfit on the training data and produce uncertainty estimates. A dropout rate of 0.5 was found to be optimum. For classification, Bayesian DenseNet-169 and Bayesian ResNet50 models with dropouts were trained with a learning rate of 10^−3^ and a batch size of 16 and 32 respectively for better generalization. We have observed that it takes around 30 epochs for the classification models to converge. Similarly 70 epochs for segmentation models were found to be sufficient for convergence. Both the classification and segmentation models were trained using Adam optimizer and binary cross entropy loss function as given in equation below [88].

$$\begin{array}{c} H_{p}(q)=-\frac{1}{C}\sum_{i=1}^{C}y_{i}\text{log}\left(p\left(y_{i}\right)\right)+\left(1-y_{i}\right)\text{log}\left(1-p\left(y_{i}\right)\right) \end{array}$$ (7)

Here *C* is the number of samples, *y*_*i*_ is truth value of the *i*^*th*^ sample and *p*(*y*_*i*_) is the probability that *i*^*th*^ sample belongs to a particular class.

### Evaluation metrics

This section discusses various evaluation metrics to validate the classification, segmentation, uncertainty estimation, and explainability methods.

#### Metrics for classification

For classification, we employ accuracy metric and F1-score as given in the equations below.

$$\begin{array}{c} Accuracy=\frac{T_{p}+T_{n}}{T_{p}+F_{p}+T_{n}+F_{n}} \end{array}$$ (8)

Here *T*_*p*_ represents True Positives, i.e, the number of samples correctly predicted as belonging to a given class. True negatives is given by *T*_*n*_, which denotes the number of samples correctly identified as not belonging to a given class. *F*_*p*_ and *F*_*n*_ denote the false positive and false negative sample predictions respectively.

F1 score is the harmonic mean of precision and recall [89]. Recall is defined as the number of true positives *T*_*p*_ over the number of true positives *T*_*p*_ plus the number of false negatives *F*_*n*_ [90] while Precision is given by the number of true positives *T*_*p*_ divided by the number of true positives *T*_*p*_ plus the number of false positives *F*_*p*_ [90]. F1−score is a more robust metric to evaluate the classification performance as it takes into consideration the class imbalance problem by giving equal importance to precision and recall, thus involving both false positives and false negatives. For classification tasks where both precision and recall are of high significance, F1-score should be maximized. F1 score ranges between 0 and 1, reaches the best value of 1 when the balance between precision and recall is perfect. F1−score is calculated as given below.

$$\begin{array}{c} F1-score=2*\frac{Precision\times Recall}{Precision∔Recall} \end{array}$$ (9)

#### Metrics for segmentation

We have employed commonly used metrics such as Dice coefficient (DI) and Jaccard index (JI) to quantify image segmentation efficiency. Both these metrics essentially measure the similarity between the ground truth and the predicted segmented image in terms of the extent of overlap between the two images. The Dice coefficient (DI) is given by

$$\begin{array}{c} DI\left(M,C\right)=2\times \frac{\left|M\cap C\right|}{\left|M|+|C\right|} \end{array}$$ (10)

The Jaccard index is given by:

$$\begin{array}{c} JI\left(M,C\right)=1-\frac{\left|M\cap C\right|}{\left|M|+|C|-|M\cap C\right|} \end{array}$$ (11)

where M represents the ground truth of segmentation, which is normally a manually-identified salient region, and C represent a mask.

#### Metrics for uncertainty

Uncertainty is measured using monte carlo dropout and test-time data augmentation. As mentioned in the above section, we calculate uncertainty *φ* but the range of these values would vary depending on the number of Monte Carlo samples. Hence we calculate normalised uncertainty *φ*_norm_ where *φ*_norm_ ∈ [0, 1] [43].

$$\begin{array}{c} \varphi_{\text{norm}}=\frac{\varphi -\varphi_{\text{min}}}{\varphi_{\text{max}}-\varphi_{\text{min}}} \end{array}$$ (12)

To split the predictions into certain and uncertain categories, we set a threshold *φ*_T_ ∈ [0, 1] where a prediction is deemed to be certain if *φ*_norm_ < *φ*_T_ and uncertain if *φ*_norm_ > *φ*_T_.

When it comes to classification, we usually end up with 4 kinds of predictions i.e incorrect-uncertain *(iu)*, correct-uncertain *(cu)*, correct-certain *(cc)*, and incorrect-certain *(ic)* predictions, where incorrect-uncertain*(iu)* refers to a prediction that was incorrect and the model was uncertain about it. Correct-uncertain*(cu)* refers to one where the model prediction is correct but the model is uncertain about it. The remaining correct-certain*(cc)* and incorrect-certain*(ic)* refer to predictions that were correct or incorrect but the mode is certain. The overall accuracy of the uncertainty estimation could be expressed as a ratio of all the desirable cases i.e correct-certain *(cc)* and incorrect-uncertain *(iu)*, and all the possible cases. This diagnostic accuracy(A) can be represented in the form [43]

$$\begin{array}{c} \text{A}(\varphi_{T})=\frac{L_{cc}+L_{iu}}{L_{cc}+L_{iu}+L_{cu}+L_{ic}} \end{array}$$ (13)

where *L* represents the count for each possible combination.

#### Metrics for explainability

Different explainability techniques like Grad-Cam, Guided Backprop, Guided Grad-Cam, and XRAI have been discussed in the previous section. To compare the performance of these techniques, we have used the bokeh effect and measured the accuracies as mentioned in [67]. The basic intuition behind this analysis is that if the above explainability techniques identify important pixels to the model’s prediction, then the model’s output of the original image and reconstructed image must go hand in hand [67]. Therefore, the bokeh effect is used to reconstruct the image, in which initially the original image is blurred and the important pixels given by the explainability techniques are added. This is done for the entire test set. Later the resultant images are passed through the classification model. The explainability techniques used are thus compared using the prediction accuracy of the classification algorithm on these reconstructed images.

## Results

In this section, we analyse the different parts of the SkiNet framework to demonstrate its efficacy. We emphasize the use of our framework using incremental experiments in order to justify its use. The following experiments have been performed:

**Experiment 1**: **Comparative analysis of segmentation techniques for preprocessing**

**Fig 5:**
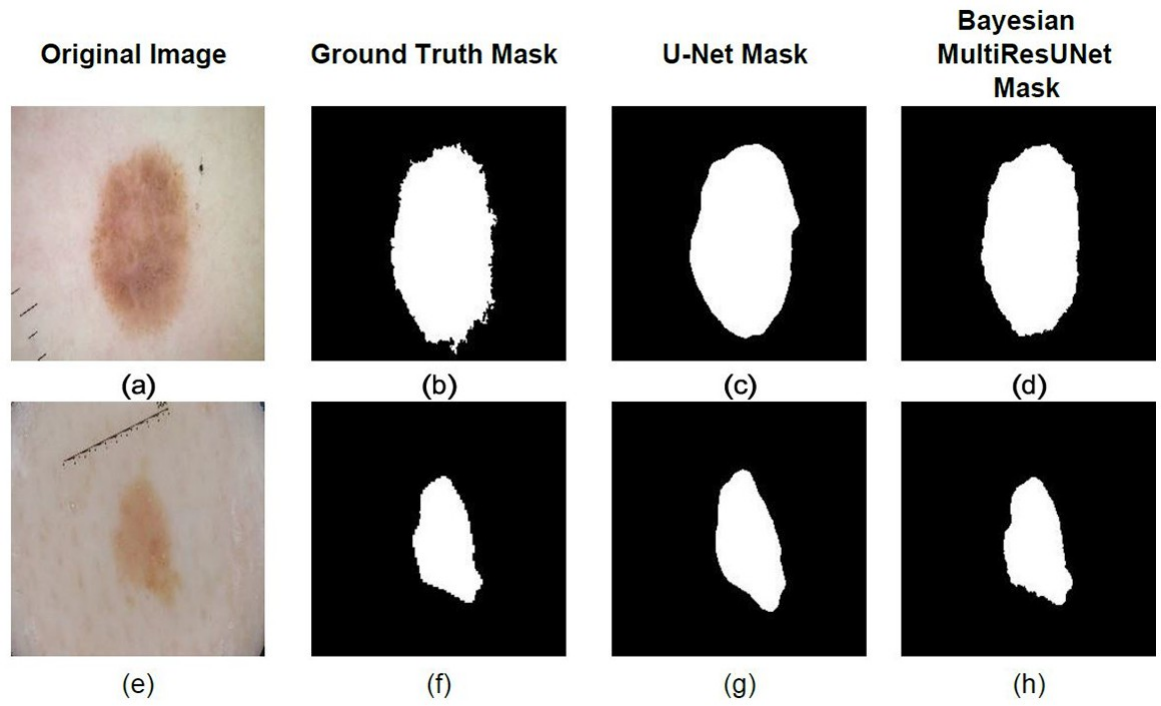
We clearly observe the Bayesian MultiResUNet outperform the U-Net with far more precise boundaries.

As observed in [33], the U-Net architecture appears to be the most effective when compared to other traditional segmentation architectures. The MultiResUnet architecture as observed in [77], was developed on the U-Net and demonstrated better effectiveness especially in the area of medical image processing. We have therefore trained Bayesian versions of MultiResUNet and U-Net. As observed in Table 1, the Bayesian MultiResUnet has outperformed other segmentation models. Hence we incorporated the Bayesian MultiResUnet as a part of the segmentation process for our SkiNet framework. Fig 5 corroborates the results as shown in Table 1.

**Table 1:** Comparitive study of various segmentation models on the ISIC 2018 Task-1 dataset.

Model	Dice Coefficient	Jaccard Index
U-Net	0.813	0.734
Bayesian U-Net	0.846	0.760
MultiResUNet	0.844	0.759
Bayesian MultiResUNet	0.852	0.767

**Fig 6:**
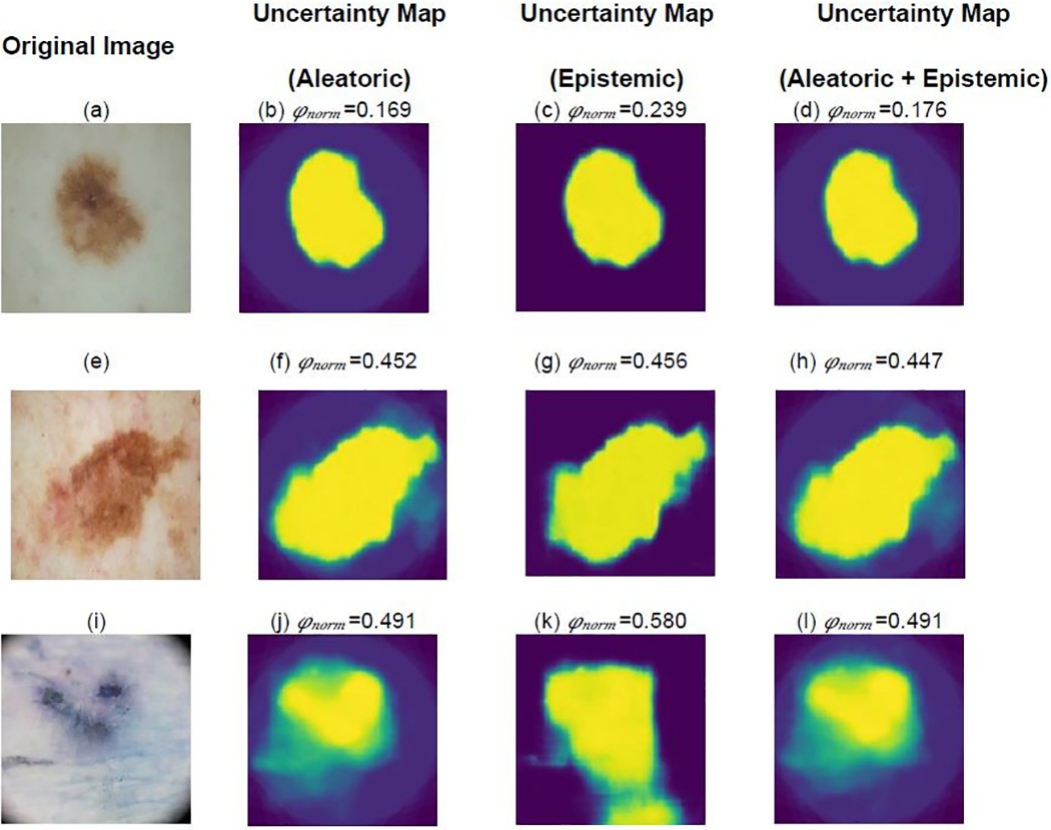
Uncertainty estimation of the Bayesian MultiResUNet.

As observed in Fig 6, the light greenish black region represents the uncertain region in our segmentation map. From Fig 6, we observe that the segmentation map produced for image Fig 6(a)is certain as aleatoric, epistemic and combined uncertainty values are well within the defined threshold. Moreover, Fig 6(b)–6(d)convey the same as the uncertain region is less in these segmentation maps. The same cannot be said regarding the maps produced for images Fig 6(e) and 6(i) as all uncertainty values are higher than the uncertainty threshold, the same can also be observed in the uncertainty maps Fig 6(f)–6(l)as significant region is highlighted as uncertain. We could also observe that the combined uncertainty maps look quite similar to that of the aleatoric uncertainty maps and that the combined uncertainty score is close to the aleatoric uncertainty score. Hence we could say that the aleartoric uncertainty has a greater contribution to the uncertainty in segmentation map.

**Experiment 2**: **Comparative analysis of classification techniques**

As observed in Table 2, The ResNet-50 and DenseNet-169 architectures perform better than other classical CNN architectures. Hence, we trained using the Bayesian versions of these architectures in order to estimate model uncertainty and classify the image. From Table 3, it can be observed that the Bayesian DenseNet-169 performs well on the ISIC-2018 dataset when compared to Bayesian ResNet-50. The McNemar test has been performed on the Bayesian ResNet50 and Bayesian DenseNet169 models to test the statistical difference between models. And the result of the experiment was statistic = 82.00 and *p*− *value*= 0.000, since p-value is less than 0.05 we reject the null hypothesis and conclude that there is a statistically significant difference between the two models. Hence Bayesian DenseNet is the better model. We therefore incorporate this classification model, as a part of our SkiNet architecture. Further, we studied the performance of the Bayesian DenseNet-169 model over each lesion category and demonstrated the accuracy and F1-score for the same in Table 4.

**Table 2:** Comparitive study of classification models on the ISIC 2018 dataset.

Model	Prediction Accuracy(%)
ResNet-50	84.87
DenseNet-169	86.67
VGG19	80.18
Xception	83.41
Inception	82.86
ResNet-50 [43]	80.45
DenseNet-169 [43]	81.35

**Table 3:** Comparitive study of Bayesian versions of top two classification models on the ISIC 2018 dataset.

Model	Prediction Accuracy(%)
Bayesian ResNet-50	85.13
Bayesian DenseNet-169	87.35
Bayesian ResNet-50 [44]	82.37
Bayesian DenseNet-169 [44]	83.59

**Table 4:** Class wise performance of Bayesian DenseNet-169.

Class	Accuracy(%)	F1-score
MEL	85.13	0.84
NV	90.66	0.89
BCC	90.82	0.91
AK	93.38	0.94
BKL	84.78	0.86
DF	89.9	0.92
VASC	90.51	0.90

**Fig 7:**
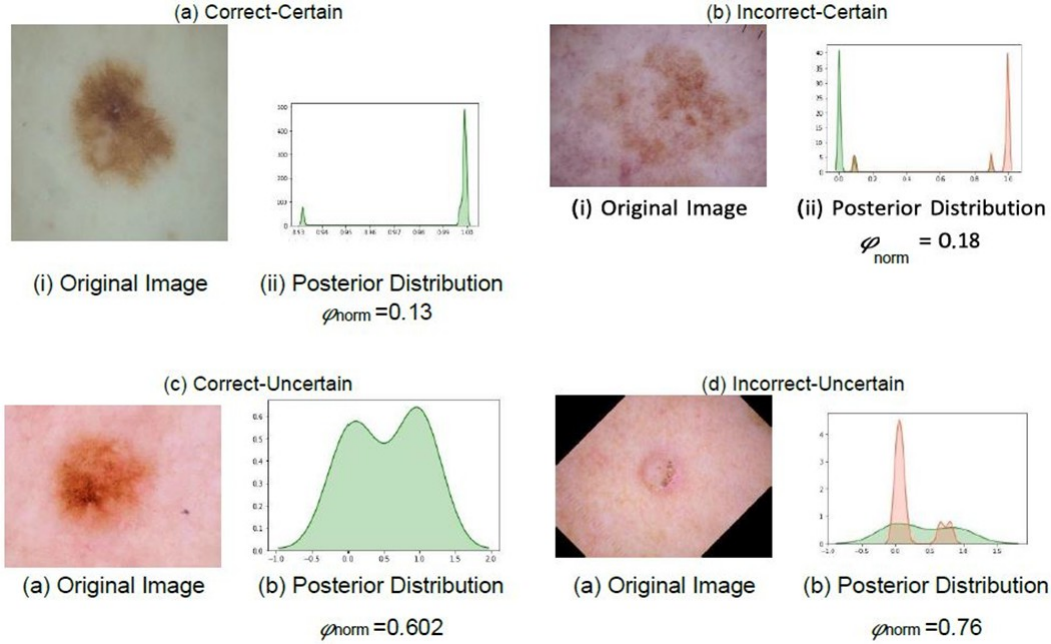
Posterior probability distributions for each of the possible scenarios i.e incorrect-uncertain *(iu)*, correct-uncertain *(cu)*, correct-certain *(cc)*, and incorrect-certain *(ic)*. Assuming that the combined *φ*_*T*_is 0.35, the red region indicates the posterior probability distribution for the incorrect class where as the green region indicates the posterior probability distribution of the correct class.

Examples of posterior probability distributions for each category discussed in the **Methods**section could be observed in Fig 7.

**Experiment 3**: **Which uncertainty type has higher impact on model performance?**

The uncertain and misclassified images are examined for various uncertainty strategies on the best model i.e Bayesian DesneNet-169 to see which sort of uncertainty has the most impact on model prediction. Table 5 shows that 209 of the 946 aleatoric uncertain images are misclassified, accounting for 22% of the total aleatoric uncertain images. To put it another way, when an image is aleatoric uncertain, there’s a 22% risk that it will be misclassified. Similarly, there is a 36% and 19.6% likelihood of being misclassified for epistemic and combined uncertain images, respectively. This obeservation is useful in a variety of medical circumstances, especially when the ground truth is unavailable. As a result, epistemic uncertainty has a greater influence on a model’s decision.

**Table 5:** Comparison of different uncertainty types.

Type	Uncertain images	Misclassified images
Aleatoric	946	209
Epistemic	251	91
Combined	1030	202

**Experiment 4**: **Comparative analysis of different explainability techniques**

**Fig 8:**
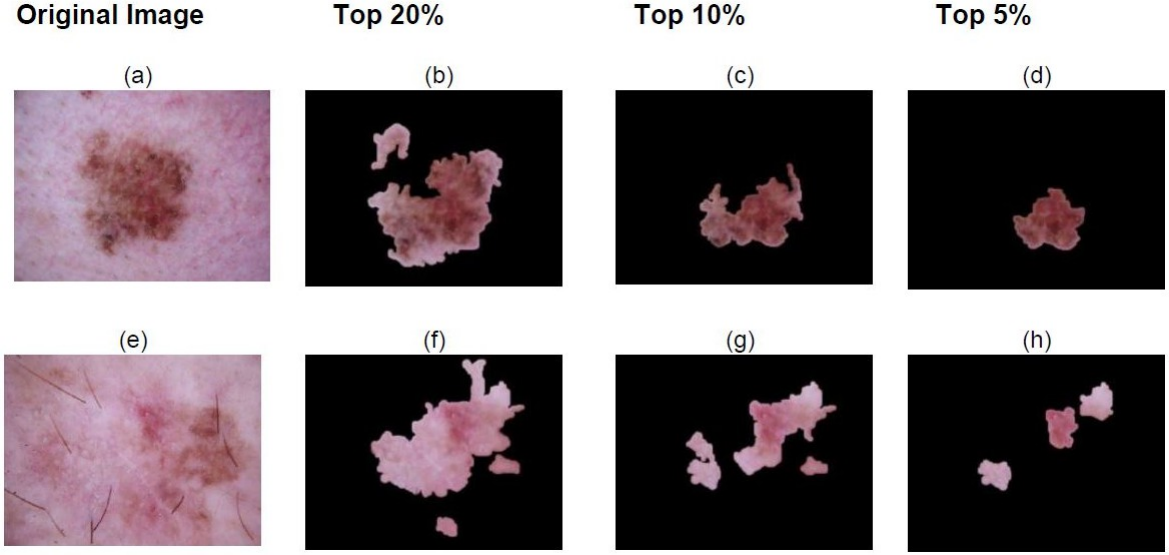
Top regions of interest identified by XRAI for classification made using the Bayesian DenseNet-169 which is part of our SkiNet framework.

**Fig 9:**
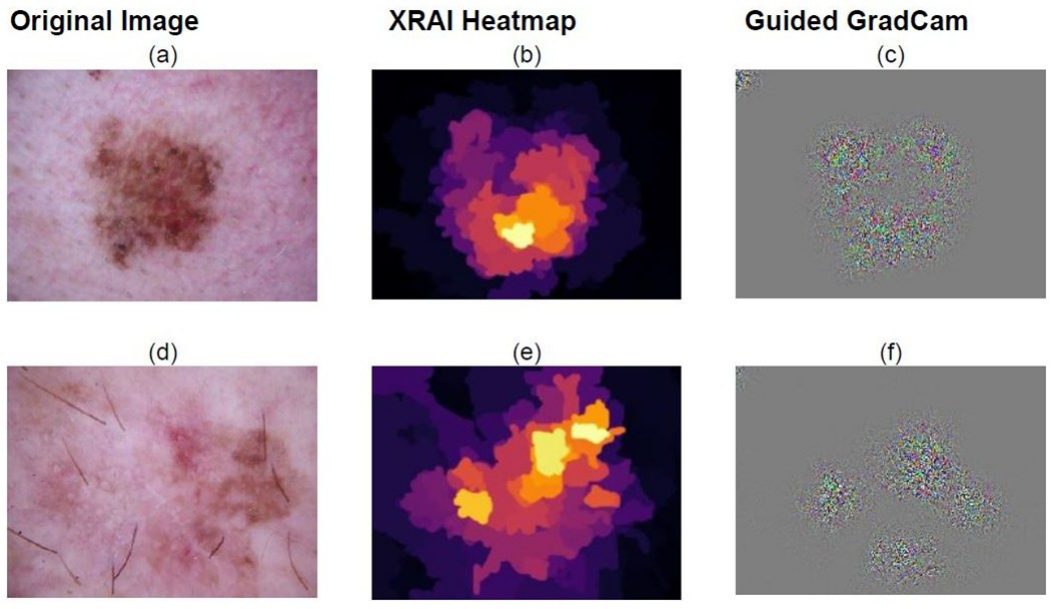
Model’s region of interest depicted by XRAI and Guided Grad CAM. For the skin lesion in Fig 9a we observe that the bottom right part of the skin lesion depicted in the XRAI heatmap in Fig 9b and Guided Grad CAM map in Fig 9c is of importance to our model. This is clearly depicted by the top 10% and top 5% plots in Fig 8c and 8d, showing that the model was heavily influenced by the dark red region. From Fig 9e, we observe that the top right part of the lesion is of importance to the model. Fig 8g and 8h show that the model is influenced by the reddish pinkish region present in the top right part of the skin lesion.

The explainability techniques as discussed previously has been compared and the result is depicted in Table 6. It is clearly visible from the Table 6 that XRAI provides a more clear visualisation of what our classification algorithm is learning. Hence we conclude that XRAI would be the best fit for the SkiNet framework and thus provide the best possible explanation behind the prediction. From Figs 8 and 9, it can be observed that XRAI would be a better method aesthetically too in order to clearly explain the reason behind a particular classification.

**Table 6:** Comparative analysis between different explainability techniques.

Explainability Technique	Accuracy
GradCam	73%
Guided Backprop	73%
Guided GradCam	77%
XRAI	84%

**Experiment 5**: **SKiNet framework performance**

Using data of Table 7 and putting in Eq 13, we clearly observe that SkiNet has a better overall diagnostic accuracy(A) of 73.65% when compared to the 70.01% of the stand-alone Bayesian DenseNet-169. It also performs better in terms of prediction accuracy(Eq 8) with an accuracy of 88.46% when compared to the 87.35% of the Bayesian DenseNet-169 as seen in Table 3.

**Table 7:** Categorical segregation of predictions made on our test data.

Category	Stand-alone Bayesian DenseNet-169	SkiNet Pipeline
Correct Certain(cc)	1602	1727
Correct Uncertain(uc)	722	627
Incorrect Certain(ic)	76	74
Incorrect Uncertain(iu)	261	233

## Discussion

**Fig 10:**
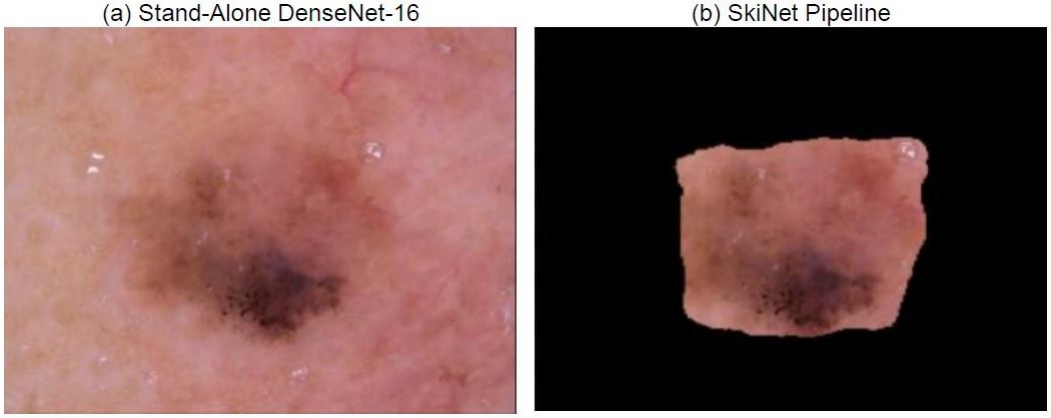
Image received by the Classification Algorithm (a) Image passed into stand-alone DesNet-169 (b) Image passed after segmentation step of SkiNet framework.

**Fig 11:**
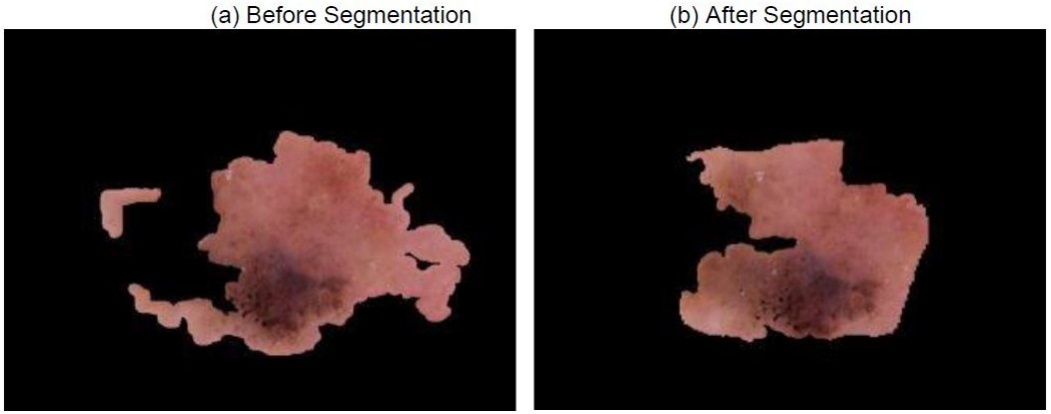
Regions of Interest identified by XRAI a.) Before Segmentation b.) After Segmentation.

Sometimes, though a prediction maybe correct, it may be deemed as uncertain due to the high uncertainty which is mainly caused by the presence of noise in the image. In the case of dermoscopic images, it is mainly in the form of sweat droplets, hair, other lesions etc. This noise could be reduced with the use of segmentation which would crop the unnecessary part out and highlight the main region of the lesion. This phenomenon is clearly observed in Fig 10, where sweat droplets and the unnecessary background is cropped out by the Bayesian MultiResUNet present in the SkiNet pipeline. This improvement can be distinctly observed in the XRAI map in Fig 11 where we clearly see that the algorithm now focuses on the lesion itself rather than the unnecessary background. From Table 8, we observe that the uncertainty drop from 0.68 to 0.30 which is within the empirically calculated threshold *φ*_*T*_of 0.35 thus leading to a certain prediction.

**Table 8:** Comparitive analysis between the performance of a Stand-alone DenseNet-169 and the SkiNet pipeline of image in above figure [CU →CC].

	Stand-Alone DenseNet-169	SkiNet Pipeline
**Ground Truth**	MEL	MEL
**Prediction**	MEL	MEL
**Uncertainty**	0.68	0.30
**Category**	Correct Uncertain	Correct Certain

**Fig 12:**
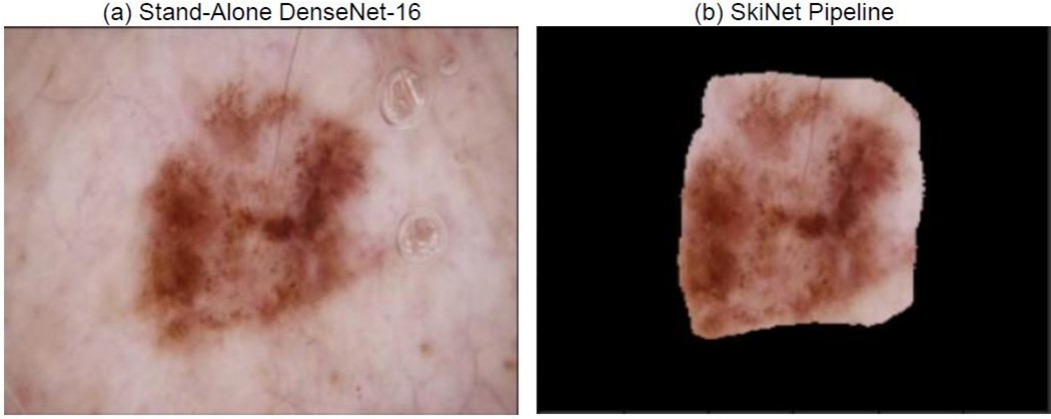
Image received by the Classification Algorithm (a) Image passed into stand-alone DesNet-169 (b) Image passed after segmentation step of SkiNet framework.

**Fig 13:**
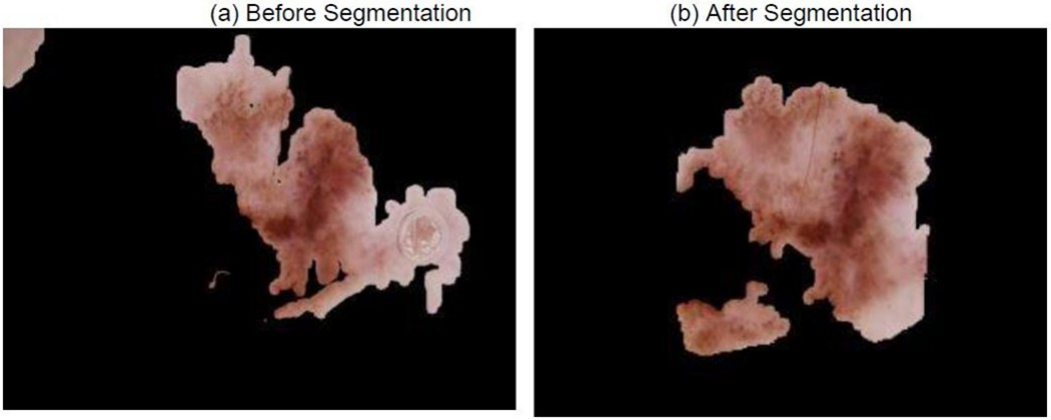
Regions of Interest identified by XRAI a.) Before Segmentation b.) After Segmentation.

Similarly, in Fig 12, the SkiNet pipeline has eliminated the water droplets in the segmentation step which changed its prediction from Incorrect Uncertain to Correct Certain as observed in Table 9. This also helps the XRAI to identify salient regions instead of focusing on droplets as shown in Fig 13.

**Table 9:** Comparitive analysis between the performance of a Stand-alone DenseNet-169 and the SkiNet pipeline [IU →CC] of above image.

	Stand-Alone DenseNet-169	SkiNet Pipeline
**Ground Truth**	MEL	MEL
**Prediction**	NV	MEL
**Uncertainty**	0.45	0.04
**Category**	Incorrect Uncertain	Correct Certain

**Fig 14:**
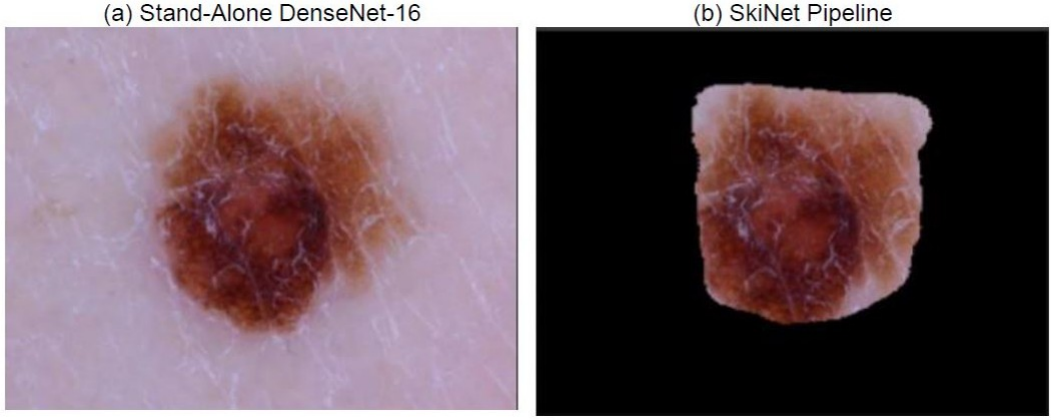
Image received by the Classification Algorithm (a) Image passed into stand-alone DesNet-169 (b) Image passed after segmentation step of SkiNet framework.

**Fig 15:**
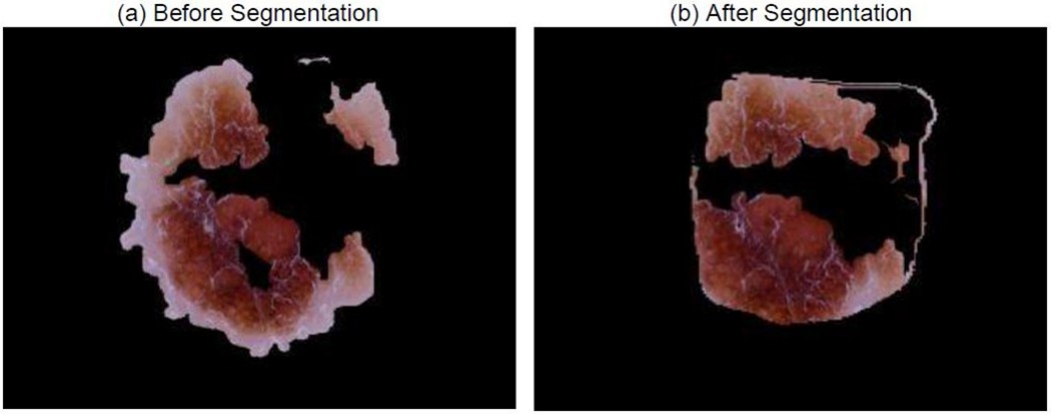
Regions of Interest identified by XRAI a.) Before Segmentation b.) After Segmentation.

From Fig 14 we observe that the Bayesian MultiResUNet present in the first step of our pipeline helps in enhancing the lesion of the image and supports in cropping out the unnecessary background. In Fig 15, we observe that the area of interest for the classification algorithm rather remains similar before and after segmentation but with expulsion of the unnecessary background which helps the algorithm to make a better prediction. Thus leading to an accurate certain prediction as demonstrated by Table 10.

**Table 10:** Comparitive analysis between the performance of a stand-alone DenseNet-169 and the SkiNet pipeline of above image [IC →CC].

	Stand-Alone DenseNet-169	SkiNet Pipeline
**Ground Truth**	MEL	MEL
**Prediction**	NV	MEL
**Uncertainty**	0.34	0.12
**Category**	Incorrect Certain	Correct Certain

## Conclusion

This article addresses the need to integrate explainability and uncertainty modeling in the automated skin lesion diagnosis process. Our study shows that UNet and Multi ResUNet have superior performance over other off-the-shelf segmentation architectures. We have therefore trained the UNet and Multi ResUNet and their bayesian versions. We have also trained various off-the-self classification models, and our experimentation shows that ResNet-50 and DesneNet-169 have superior performance comparatively, hence we have trained their bayesian verisons. Finally we conclude that Bayesian MultiResUNet, DenseNet 169 are the best models for segmentation and classification respectively. In this paper, we have proposed a novel SkiNet pipeline for the diagnosis of skin lesion. The proposed Bayesian Multi ResUNet which is used for segmentation, also produces uncertainty maps to incorporate the confidence measure. The DenseNet-169 with added dropout has been used for classification and has demonstrated superior performance over the original. The addition of segmentation as a pre-processing step for classification has greatly helped the efficiency of the classification model. The uncertainty score of the segmentation model’s output is used to pass only the most confident predictions to classification model. The uncertainty score of the classification model tests the confidence of the model’s prediction and suggests second opinion in the event of less positive predictions thereby reducing misdiagnosis to some degree. The diagnostic accuracy of stand-alone Bayesian DenseNet-169 is 70.01%, which further improved to 73.65% after performing segmentation using the proposed SkiNet pipeline. When deploying such models, one could use model explanations to “gate” the use of the machine learning system. To build trust of the medical community in the proposed model, we use an explainability map that shows the salient region for the model. Using the saliency maps provided by various techniques such as GradCAM, Guided Backprop, Guided GradCAM and XRAI, the original images are reconstructed with the aid of Bokeh effect. They are then passed through the classification model and the accuracy scores thus obtained clearly demonstrate a superior performance of XRAI with an enhanced 84% accuracy. The results of the proposed pipeline is quite encouraging and can be generalized for other similar tasks in the medical domain. This article has used post-hoc interpretability methods however, we would also like to explore some pre-hoc interpretation methods like attention mechanism while training the model in order to further enhance the model’s performance.
